# Applying Modified VP53A Recombinant Protein as an Anti-White Spot Syndrome Virus Biological Agent in *Litopenaeus vannamei* Farming

**DOI:** 10.3390/v14071353

**Published:** 2022-06-21

**Authors:** Jeff Chia-Kai Hsu, Huai-Ting Huang, Han-Jia Lin, Hsin-Yiu Chou, Po-Yu Huang, Anuphap Prachumwat, Li-Li Chen

**Affiliations:** 1 Institute of Marine Biology, National Taiwan Ocean University, Keelung 20224, Taiwan; jeffhsu@innocreatebio.com; 2 Innocreate Bioscience Co., Ltd., Zhonghe District, New Taipei City 23557, Taiwan; 3 Department of Aquaculture, National Taiwan Ocean University, Keelung 20224, Taiwan; twinkleqazwsx784@gmail.com (H.-T.H.); hychou@mail.ntou.edu.tw (H.-Y.C.); 4 Department of Bioscience and Biotechnology, National Taiwan Ocean University, Keelung 20224, Taiwan; hanjia@mail.ntou.edu.tw; 5 Department of Life Science, National Taitung University, Taitung 95092, Taiwan; abcm0113@nttu.edu.tw; 6 Aquatic Animal Health Research Team, Integrative Aquaculture Biotechnology Research Group, National Center for Genetic Engineering and Biotechnology (BIOTEC), National Science and Technology Development Agency (NSTDA), Pathum Thani 12120, Thailand; 7 Center of Excellence for Shrimp Molecular Biology and Biotechnology (Centex Shrimp), Faculty of Science, Mahidol University, Bangkok 10400, Thailand; 8 Center of Excellence for the Oceans, National Taiwan Ocean University, Keelung 20224, Taiwan

**Keywords:** shrimp, recombinant protein, immune gene, intestinal microbiota

## Abstract

Shrimp farming is an important economic activity. However, due to the spread of pathogens, shrimp aquaculture is becoming increasingly difficult. Many studies have confirmed that white spot syndrome virus (WSSV) recombinant proteins can inhibit viral infection. Among them, VP53 recombinant protein has been found to reduce mortality upon WSSV challenge. This study was conducted in Kaohsiung, Taiwan and reports the first field feeding trial to demonstrate that WSSV recombinant proteins can improve shrimp survival rates at a farming scale. Prior to the feeding trial, the shrimp were confirmed to be slightly infected with WSSV, *Vibrio parahaemolyticus* strains causing acute hepatopancreatic necrosis disease (AHPND), non-AHPND *V. parahaemolyticus* strains, and *Enterocytozoon hepatopenaei* (EHP), which are common pathogens that shrimp farmers often face. The shrimp were then divided into two groups: a control group (C group) fed with a commercial diet and a protein group (P group) fed with the same commercial feed with VP53 recombinant protein. Our findings indicated that the survival rate and expression of immune genes of the P group were higher than those of the C group. The intestinal microbiota of the two groups were also analysed. Collectively, our results confirmed that the recombinant WSSV envelope protein derivative can be used as an effective anti-virus biological agent in shrimp farms.

## 1. Introduction

Shrimp farming is an important economic activity and provides a quality protein source for human consumption [1]. However, the shrimp industry has been facing challenges due to pathogen outbreaks [2,3]. Among these, the white spot syndrome virus (WSSV) causes the most serious shrimp viral disease, resulting in huge economic losses in the shrimp farming industry worldwide. WSSV has a wide host range and causes 100% mortality within 7 days of infection in cultured penaeid shrimp [4]. In fact, the Taiwan shrimp aquaculture industry has not yet recovered from the first WSSV outbreak in 1992 [5,6].

WSSV is a large, double-stranded DNA virus with enveloped virions of oval shape, approximately 120 nm in diameter and 275 nm in length [4,7]. Because WSSV is an enveloped virus, it can invade the host through direct virus–host interactions [8]. The structure of WSSV is complex with at least 58 structural proteins, of which at least 30 are viral envelope proteins [9,10,11,12,13]. Among these envelope proteins, VP53A contains a domain similar to the lectin α-chain of legumes [14]. This protein can interact with the chitin-binding protein (CBP) of the host and other viral envelope proteins of the virus, including VP24, VP31, VP32, and VP39B [14,15]. CBP may be a receptor for WSSV and has been found to be involved in the process of viral infection [16]. Therefore, the VP53A recombinant protein can significantly reduce and delay mortality upon WSSV challenge.

In shrimp ponds, the efficacy of the VP53A recombinant protein may be inhibited by many field factors such as other pathogens, fluctuating pH and temperature. Therefore, a field feeding trial with VP53A is needed to determine whether VP53A remains effective in the field for its future practical application in the shrimp industry. In this study, a field feeding trial was performed in southern Taiwan to evaluate the anti-WSSV effectiveness of Inno A1, a recombinant VP53A-based biological agent. Shrimp post-larvae were stocked in a pond for four weeks and then analysed for pathogens and growth performance before being divided into two groups: a control group (C group) fed with a commercial diet and a protein group (P group) fed with Inno A1-containing feed. The shrimp farm production, pathogen detection, immune gene expression, and the intestinal microbiota of the two groups were analysed after the 6-week feeding trial. Our study evaluated the effectiveness of a recombinant protein-based anti-WSSV agent in the field and serves as a basis for the development of better disease management strategies in shrimp farms in the future.

## 2. Materials and Methods

### 2.1. Animals and Sample Collection

*Litopenaeus vannamei* tests were performed in Linyuan, Kaohsiung, Taiwan. In August 2018, shrimp post-larvae were first cultured in indoor cement ponds (6 × 3 × 0.6 m^3^) with a water depth of 0.3 m for 4 weeks. Before shrimp culture, all ponds were treated with potassium permanganate for several days. At the end of the fourth week before starting the feeding trial, shrimp were collected and noted as the B group. Then, 4000 shrimp with an average body length of 5 ± 0.5 cm and an average weight of 1 ± 0.2 g were randomly divided into two groups in two separate cement ponds of the same size (6 × 3 × 0.6 m^3^). During the six-week feeding trial, the shrimp were fed four times daily with commercial feed (C group) or Inno A1-containing feed (P group).

### 2.2. Recombinant Protein Preparation

The recombinant protein, Inno A1, derived from VP53A was prepared by Innocreate Bioscience Co., Ltd. (New Taipei City, Taiwan). In short, recombinant Inno A1 protein was generated by an *Escherichia coli* expression system. Pilot-scale fermentations were performed using 10 L stirred-tank reactors (Micro-Giant BioEngineering, Taichung City, Taiwan). After fermentation, cells were harvested by centrifugation at 4000 rpm for 60 min at 4 °C, followed by disruption using a high-pressure cell disruptor (Pressure BioSciences, Inc., South Easton, MA, USA). The target protein was purified by a tangential flow filtration (TFF) system (Spectrum, Brockton, MA, USA), and a Ni-NTA purification system (QIAGEN). To avoid endotoxin contamination, the Triton X-114 phase separation method used in the Ni-NTA purification system can remove endotoxin [17,18]. The purified protein was then sent to Energenesis Biomedical Co., Ltd. (Taipei City, Taiwan) for further characterisation by high performance liquid chromatography (HPLC) and liquid chromatography-tandem mass spectrometry (LC-MS/MS).

### 2.3. Preparation of Test Diets

The basal diet was a commercial shrimp feed (Tung−Li Feed Industrial CO., LTD., Pingtung County, Taiwan). For coating, the recombinant protein solution was mixed with the feed and incubated at room temperature for 15 min to allow for the absorption of recombinant protein solution to prevent dispersion of the recombinant protein in water. The recombinant protein-containing feed was then dried at 40 °C overnight and stored at 4 °C until administered to the shrimp.

### 2.4. Pathogen Detection

Major shrimp pathogens including WSSV, *Vibrio parahaemolyticus,* which cause acute hepatopancreatic necrosis disease (AHPND), general *V. parahaemolyticus* (i.e., *V. parahaemolyticus* without toxin genes), and *Enterocytozoon hepatopenaei* (EHP) were monitored. WSSV QD, AHPND QD (detected through both toxin genes and *V. parahaemolyticus*), and EHP QD (Innocreate Bioscience, New Taipei City, Taiwan) qPCR-based pathogen detection kits were used. The reactions were performed using an ABI 7900HT Fast Real-Time PCR System (Thermo Fisher Scientific, Waltham, MA, USA) according to the manufacturer’s instructions.

### 2.5. Haemolymph Collection, RNA Extraction, and First-Strand cDNA Synthesis

The collection of haemolymph from shrimp was performed with a 1 mL syringe containing cold anti-coagulant (27 mM sodium citrate, 336 mM NaCl, 115 mM glucose, and 9 mM EDTA, pH 7.0) with a 25G × 5/8″ needle. The haemolymph solution was centrifuged at 1000× *g* for 5 min and the supernatant was removed. The total RNA of the haemocytes was extracted with the TRIzol reagent (Thermo Fisher Scientific, Waltham, MA, USA) according to the manufacturer’s instructions. The haemocytes were lysed with 400 μL TRIzol reagent. The mixture was subjected to 2-propanol and ethanol precipitation. Total RNA in 75% ethanol was centrifuged at 12,000× *g* at 4 °C for 5 min. The pellets were resuspended in DEPC water and quantified by spectrophotometry. After RNA extraction, an aliquot of 1 μg RNA was treated with 1 U of RNase-free deoxyribonuclease I (DNase I) (Thermo Fisher Scientific, Waltham, MA, USA) at 25 °C for 30 min, after which the reaction was stopped with EDTA at 65 °C for 10 min. The first-strand cDNA was synthesised by the addition of 5 μL 10 × First-Strand buffer, 50 μm oligo dT-anchor primer (Roche), and 1 μL of 10 μm dNTP mixture, 2 μL 0.1 M DTT, 1 μL 40 U RNase inhibitor (Thermo Fisher Scientific, Waltham, MA, USA), and 1 μL HiScript I reverse transcriptase (Bionovas, Toronto, ON, Canada). The reaction proceeded at 42 °C for 60 min and was terminated at 75 °C for 15 min. cDNA samples were used in subsequent gene expression analyses by quantitative real-time PCR.

### 2.6. Gene Expression by Quantitative Real-Time PCR Analysis

The primers for gene expression by quantitative real-time PCR (qPCR) in this study (Table 1) were described in Wang et al. [19], Leu et al. [20] and Chiang et al. [21], and those of two additional genes were designed for better specificity. QPCR reactions with the haemocyte cDNA samples as templates were conducted using the Fast SYBR Green Master Mix (Thermo Fisher Scientific, Waltham, MA, USA) and performed using the ABI 7900HT Fast Real-Time PCR System (Thermo Fisher Scientific, Waltham, MA, USA). The thermal cycling program was 2 min at 50 °C, 10 min at 95 °C, and 45 cycles of 15 s at 95 °C and 20 s at 60 °C. Dissociation curve analysis was included to examine the specificity of the qPCR products. Gene expression analysis was conducted via the 2(-Delta Delta Ct) method according to Livak and Schmittgen [22].

### 2.7. DNA Extraction and High-Throughput Amplicon Sequencing

The total DNA of the shrimp gut, pond water and sediment samples was extracted using the TANBead Tissue Total DNA kit (Taiwan Advanced Nanotech, Taoyuan City, Taiwan) on a Maelstrom automated extraction system (Taiwan Advanced Nanotech, Taoyuan City Taiwan) according to the manufacturer’s instructions. DNA concentration was determined using a Nano-300 spectrophotometer (Clubio, Taoyuan City Taiwan). Sequencing libraries were generated according to the 16S Metagenomic Sequencing Library Preparation protocols (Illumina, San Diego, CA, USA) for the V3-V4 region of the 16S rRNA gene (Table 1). Amplicons were confirmed by gel electrophoresis, purified by Agencourt AMPure XP (Beckman Coulter, Indianapolis, IN, USA) and quantified using the Qubit dsDNA HS Assay Kit (Thermo Fisher Scientific, Waltham, MA, USA). The purified V3-V4 target amplicon was used for downstream analyses, and PCR products were also purified as described above. Library quantification was conducted using three methods, including the Qubit dsDNA HS Assay kit (Thermo Fisher Scientific, Waltham, MA, USA), the KAPA Library Quantification kit (Roche Sequencing and Life Science, Wilmington, MA, USA), and the Qsep100 Bio-Fragment Analyser (BiOptic, New Taipei City, Taiwan). Libraries were sequenced using the Illumina MiSeq Reagent Kit v3 (600-cycle) (Illumina, San Diego, CA, USA) by Seeing Bioscience Co., Ltd. (New Taipei City, Taiwan).

### 2.8. Microbiota Analysis

Amplicon sequence variant (ASV) construction was performed with QIIME2 (version 2019.7.0) [23] using the DADA2 denoising algorithm [24] and truncated lengths of 275 and 235 base pairs for forward and reverse sequencing reads, respectively, on the cleaned reads produced by removing primer sequences of the raw reads with Cutadapt (https://cutadapt.readthedocs.io/ (accessed on 16 May 2020)). These procedures were used to produce a set of ASVs that were imported into R and filtered for ASVs found in ≥2 samples and of ≥0.1% abundance for the analysis of alpha and beta diversities and differential abundance among the groups. A compositional data (CoDa) analysis approach [25] was used to generate principal component analysis (PCA) plots and for differential abundance tests with the ALDEx2 v1.6.0 Bioconductor package using significantly abundant ASVs of an expected effect size difference of ≥1 [26]. A standard count data analysis with phyloseq [27] and microbiome (http://microbiome.github.com/microbiome (accessed on 15 October 2020)) packages were used for alpha diversity index calculation and non-metric multidimensional scaling (NMDS) with a Bray–Curtis dissimilarity distance. Additional graphs were plotted with the ggplot2 (https://ggplot2.tidyverse.org (accessed on 29 May 2020)) and ggpubr (https://rpkgs.datanovia.com/ggpubr/ (accessed on 16 May 2020)) packages. The amplicon sequencing reads were deposited in the Short Read Archive database of the National Center for Biotechnology Information (NCBI) database under Accession Number PRJNA735052.

## 3. Results

### 3.1. Preparation of VP53A Derived Recombinant Protein, Inno A1

**Figure 1:**
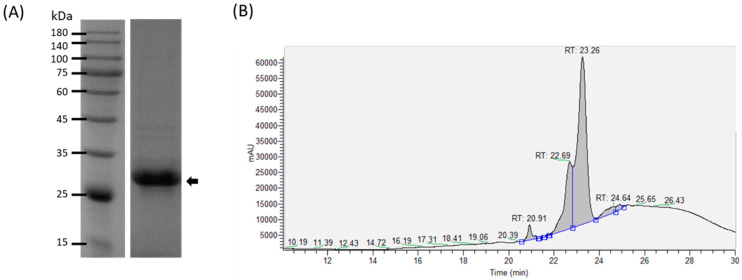
Purification and identification of Inno A1. (**A**) SDS-PAGE was used to determine the recombinant protein generated by *Escherichia coli*. The arrow indicates Inno A1. (**B**) High-performance liquid chromatography (HPLC) chromatogram of the purified product.

The VP53A-derived recombinant protein, Inno A1, was produced with an *Escherichia coli* expression system, the molecular weight of the protein was approximately 28 kDa (Figure 1A). The recombinant protein was purified, identified, and quantified by high-performance liquid chromatography (HPLC) and liquid chromatography-tandem mass spectrometry (LC-MS/MS) (Figure 1B). The major protein Inno A1 accounted for approximately 92% of the purified product.

### 3.2. Administration of Inno A1-Containing Feed Increases Shrimp Production and Survival Rates

At the fourth week of the culture before the feeding trial start, the health status of 14 randomly selected 1 g shrimp post-larvae was evaluated (B group; see Materials and Methods). Among these B group shrimp samples, a few shrimp (2 to 4 samples) tested positive for AHPND *V. parahaemolyticus*, non-AHPND *V. parahaemolyticus*, and WSSV. Moreover, EHP was detected in the majority (13) of the samples (Table 2). Four thousand B-group shrimp were divided into groups C and P (2000 shrimp each), which were fed four times daily with commercial feed and Inno A1-containing feed, respectively. After the six-week feeding trial, far fewer surviving shrimp were observed in the C group than in the P group (43 vs. 1111 shrimp, respectively). Indeed, the average body length and weight of the P group shrimp were higher than those in the C group (Table 3). Among the harvested shrimp, a large proportion was infected with EHP in both the P and C groups; however, non-AHPND *V. parahaemolyticus* was detected in only one sample in the C group but none in the P group. Moreover, WSSV and AHPND *V. parahaemolyticus* were not detected in either group (Table 2).

### 3.3. Inno A1 Can Induce the Expression of Immune-Related Genes in Shrimp

**Figure 2:**
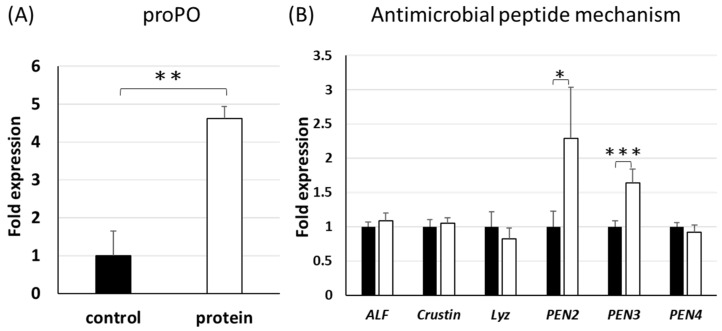
Innate immune gene expression of shrimp haemocytes. (**A**) *proPO* gene; (**B**) antimicrobial peptide genes. The black and white bars represent the C group and the P group, respectively. Each bar represents the mean ± SD. Statistical significance was calculated using Student’s *t*-test. Significant differences between the compared groups are indicated with * (0.005 ≤ *p* < 0.05), ** (0.0005 ≤ *p* < 0.005) and *** (*p* < 0.0005).

**Figure 3:**
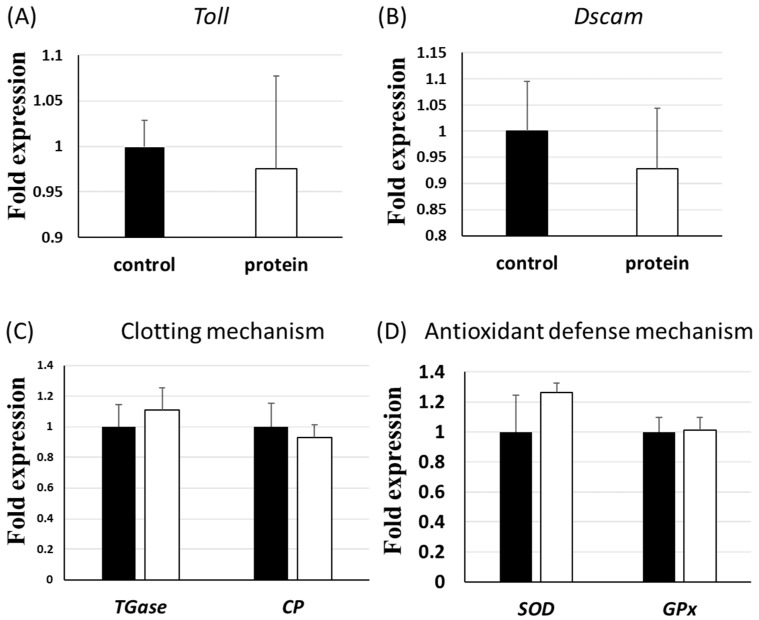
Innate immune gene expressions of shrimp haemocytes. The black and white bars represent the C group and the P group, respectively. Each bar represents the mean ± SD.

Shrimp innate immune responses are important for shrimp health [17]. Here, the expression of innate immune gene groups was determined. The *prophenoloxidase* (*proPO*) gene of the *proPO* activating system, which is considered a major innate defence system of shrimp, was up-regulated in the P group compared to the C group (Figure 2A). Antimicrobial peptides (AMPs) also constitute the first line of defence against pathogens in shrimp, and several AMP genes such as penaeidin 2 (*PEN2*) and penaeidin 3 (*PEN3*) were up-regulated in the P group compared to the C group (Figure 2B). Furthermore, there was no significant change in the expression of other investigated immune genes associated with the antimicrobial immune response (*Toll*), shrimp Down syndrome cell adhesion molecules (*Dscam*), haemolymph clotting mechanisms (transglutaminase (*TGase*) and clotting protein (*CP*), antioxidant defence mechanisms (superoxide dismutase (*SOD*) and glutathione peroxidase (*GPx*)), and the antimicrobial peptide system (anti-lipopolysaccharide factors (*ALFs*), crustin, lysozymes (*Lyz*), and penaeidin 4 (*PEN4*)) (Figure 2B and Figure 3).

### 3.4. Effects of Inno A1 Dietary Supplementation on the Intestinal Microbiome of Shrimp

**Figure 4:**
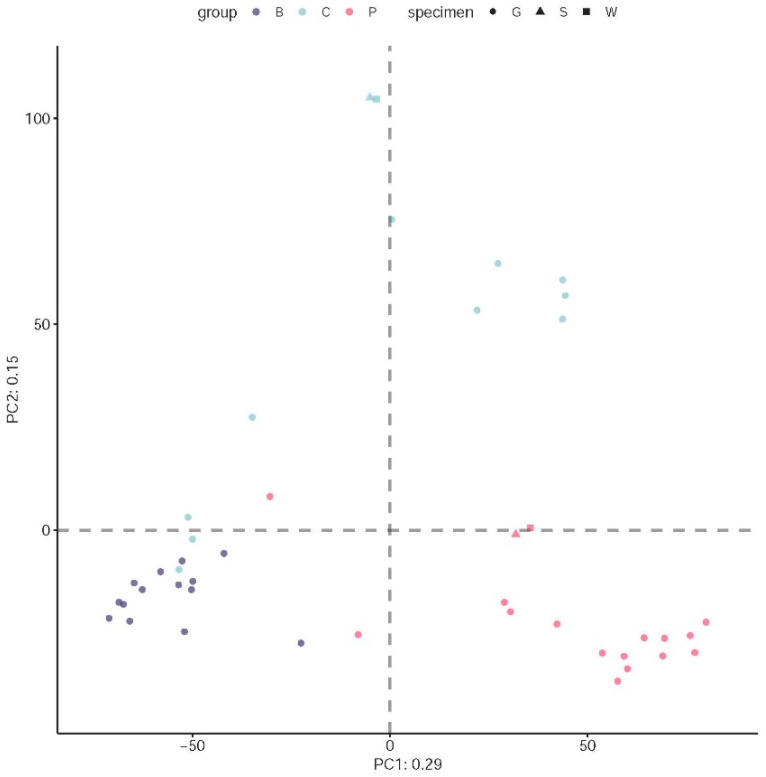
Compositional PCA plot of samples for the ASV dataset. Each point is a sample [B group (purple), C group (green), and P group (red)] and the distance between points is proportional to the multivariate difference between samples. The ability to directly interpret the plot is limited by the proportion of the explained variance (29% of the first component and 15% of the second component).

The microbial composition of shrimp intestines and the pond environments (water and sediment specimens) were assessed via V3-V4 16S rRNA amplicon analysis. A total of 3,333,593 quality-filtered reads out of 4,732,901 raw reads were used to construct amplicon sequence variants (ASVs) with QIIME2 denoising using DADA2. Next, 979 ASVs found in ≥2 samples and with ≥0.1% abundance were used in the subsequent analyses for alpha and beta diversities and differential abundance among the groups. Principal component analysis (PCA) revealed a separation among the bacterial profiles of the three groups of samples on the first two components. The samples of the B group tended to cluster together and separated from both the P and C groups on the first axis (29% of the variances) and the P group separated from the C group on the second axis (15% of the variances) (Figure 4). Some shrimp samples from the P and C groups (two and four samples, respectively) were closer to those of the B group. The culture water and soil sediment samples clustered together with the P and C groups. However, these samples could still be clearly distinguished from the P and C groups (Figure 4). PCA suggested that the shrimp gut bacterial profiles were different between the two culture ages (B group vs. P and C groups) and that the shrimp gut bacterial profiles of the P group were markedly different from those of the C group after the feeding trial. PCA also suggested that the bacterial compositions of the pond environments (water and soil sediment) were different between the P and C groups. This bacterial profile pattern was also supported by non-metric multidimensional scaling (NMDS; Supplementary Materials, Figure S1).

**Figure 5:**
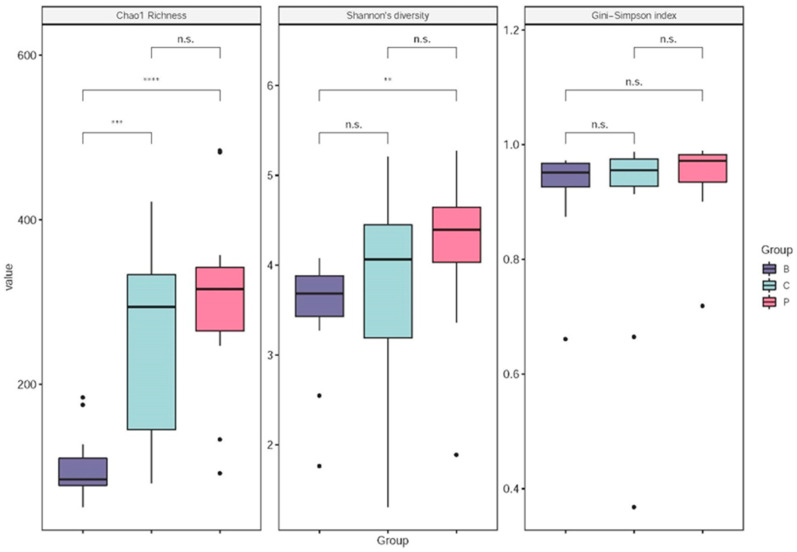
Pairwise comparisons of alpha-diversity among the B group (*n* = 14, purple), the C group (*n* = 12, green), and the P group (*n* = 17, red) according to Chao1 richness index, Shannon’s diversity index, and Gini−Simpson index. Significant differences from Mann−Whitney U tests are indicated by asterisks (n.s.: *p* ≥ 0.05; **: 0.001 ≤ *p* < 0.01; ***: 0.0001 ≤ *p* < 0.001; ****: *p* < 0.0001).

The B group also exhibited a significantly lower alpha−diversity than the P and C groups based on both the Chao1 richness and Shannon’s diversity indices (*p* < 0.01) but not with the Gini−Simpson index (*p* > 0.05; Figure 5). It is also worth noting that the P and C groups had similar alpha−diversities (Chao1 richness, Shannon’s diversity, and Gini−Simpson indices; *p* > 0.05; Figure 5).

Differential abundance analysis of bacterial profiles was used to determine bacterial ASVs associated with the P and C groups on the chloroplast-excluded ASV dataset. A total of 59 ASVs were found to be significantly more abundant in the P group than in the C group (P > C), whereas 35 ASVs were found to be significantly less abundant in the P group than in the C group (P < C) (Table 4). Among 59 P > C ASVs, the ASVs with the highest abundance fold changes (1302.7) were represented by the Flavobacteriaceae of the Bacteroidetes phylum (8 significant ASVs in this phylum), the second-highest fold change (980.9) ASV was in the Rhodobacteraceae of the *Alphaproteobacteria* (20 significant ASVs in Rhodobacteraceae; five significant ASVs with fold changes > 500), and the third-highest fold change (867.2) ASV was in Gammaproteobacteria incertae sedis of Gammaproteobacteria (6 significant ASVs in this group). Among 35 C > P ASVs, the highest abundance fold changes (914.7) were in Gammaproteobacteria and Gammaproteobacteria incertae sedis of Proteobacteria (1 ASV in this group), the second-highest fold change (647.7) ASV was in Patescibacteria (6 significant ASVs in this phylum), and the third-highest fold changes (565.5) was in Marinilabiliaceae of Bacteroidetes (2 significant ASVs in this group). Importantly, Gammaproteobacteria had the highest number of ASVs with a significantly higher abundance in C than in P, with Alteromonadales, Vibrionales, Chromatiales, and Steroidobacterales being representatives of the Gammaproteobacteria.

## 4. Discussion

In previous studies, many recombinant proteins have been found to inhibit WSSV invasion in vitro and reduce the mortality of WSSV-infected shrimp in vivo [9,15,28]. However, these experiments were only small-scale laboratory tests, and no field experiments have been conducted. VP53A is an envelope protein and plays an important role in WSSV entry. VP53A may form protein complexes with other viral proteins to infect the host [15]. In an in vivo neutralisation experiment, VP53A increased the survival rate of the shrimp challenged with WSSV by 40% [16]. Therefore, VP53A was selected for the field experiment. Here, our study is the first field study attempting to evaluate the effectiveness of recombinant proteins in a shrimp pond. In this experiment, the shrimp were divided into two groups: the C and P groups. The C group (i.e., the control group) was fed with commercial feed, whereas the P group (i.e., the experimental group) was fed with commercial feed supplemented with Inno A1, which was derived from the WSSV envelope protein VP53A. During the feeding trial period, farmed shrimp were exposed to various pathogens. A high proportion of the initial shrimp stock in this study (B group) tested positive for EHP, as well as AHPND and non-AHPND *Vibrio parahaemolyticus* isolates. A low proportion of the samples tested positive for WSSV (Table 2), which confirmed the quality of the initial stock and the overall pathogen prevalence in the test ponds. Due to the presence of these pathogens at the beginning of the experiment, the shrimp in the C group pond only had a 2.15% survival rate after the 6-week culture period (Table 3). This low survival rate was similar to ponds in the same area, as reported by many shrimp farmers. Surprisingly, a significantly higher survival rate (55.54%) as observed in the P group pond compared to the C group, which was also supported by better growth performance of the shrimp, e.g., the longer length and higher weight of the P group compared to the C group (Table 2). Such a better survival rate and performance in the P group were likely attributed to the Inno A1-supplemented diet. Therefore, we investigated the differences in the C and P groups in terms of immune gene expression and intestinal microbiota.

Three immune genes in the proPO activating system, *ProPO*, and the antimicrobial peptides (AMPs) system, *PEN2* and *PEN3*, were significantly up-regulated in the P group compared to the C group (Figure 2). The proPO activating system induces melanin formation and cytotoxic reactions, as well as encapsulation and phagocytosis against pathogens [29,30,31]. Antimicrobial peptides could exert protective roles against many pathogens, including bacteria, fungi, parasites, and viruses [32]. The *proPO*, *PEN2*, and *PEN3* genes were up-regulated in the P group that had been fed the diet containing the protein complex for six weeks, demonstrating that Inno A1, the recombinant proteins derived from VP53A, could help shrimp to fight against pathogens. EHP does not cause death but does induce growth retardation in shrimp. Even though the shrimp of the P group were still infected with EHP, the P group exhibited better growth performance and survival rates than the C group.

In addition to changes in immunity, our study also sought to determine whether the gut microbiota composition of shrimp was affected by Inno A1 treatment. Shrimp intestinal microbiomes can change and vary due to growth stages, environmental changes, pathogen infections, and food sources [33,34,35,36,37]. The differences in the intestinal bacterial profiles (PCA) and bacterial diversity (alpha diversities) of the B group shrimp from those of the P and C groups (Figure 4 and Figure 5) were likely attributable to age differences and developmental stages in the B group compared to the P and C groups (6 weeks apart). Previous studies have demonstrated that different shrimp from different growth stages have distinct intestinal microbiomes. This is likely related to their feeding behaviour and food types [38,39]. However, the observed differences in intestinal bacterial profiles between the P and C groups were likely determined by the different health status of the two groups (Figure 4). It is worth noting that the P and C groups did not exhibit a significant difference in bacterial diversity (alpha diversities; Figure 5). Our results clearly indicated that the P group pond exhibited better shrimp survival rates and higher expressions of several key immune-related genes, which suggested a better health status of shrimp in the P group than in the C group. Indeed, the C group pond experienced disease outbreaks that caused almost 100% mortality rates by the sixth week of the experiment. The mortality in the C group was likely due to some earlier detected pathogens (i.e., in the B group shrimp samples; Table 2), even though the remaining shrimp were only positive for EHP and non-AHPND *Vibrio parahaemolyticus*. According to previous studies, the intestinal flora of shrimp also changes due to the outbreak of diseases, such as AHPND. In fact, the microbiota starts to change days before an AHPND outbreak [40]. Although the shrimp in sample B did not show a large-scale response when the samples were collected, they were found to be partially infected with AHPND, WSSV, or EHP (Table 2). This may explain the fact that some shrimp samples from the C group damaged by disease outbreak exhibited similar bacterial profiles to some of group B.

Many studies have indicated that the intestinal microbiota of shrimp is associated with the health of shrimp. The B group exhibited a lower microbial diversity (Figure 5) and *Vibrionaceae* was the dominant intestinal microbial taxon in the B group. *Vibrionaceae* classified under Vibrionales in the Gammaproteobacteria class is comprised of several known opportunistic pathogens that severely affect shrimp. Therefore, the shrimps in the C group were weak and even dead. At the end of the sixth week of the feeding trial, the *Vibrionaceae* still occurred in the gut of the C group but decreased greatly in the gut of the P group (Table 4, reduced by 166.5-fold). The shrimp of the P group were healthier than those of the C group. Therefore, we speculated that this VP53A-derived protein could upregulate immune genes against opportunistic pathogens in shrimp.

So far, at least 11 WSSV envelope proteins have been confirmed to be involved in viral invasion, including VP24, VP32, VP39B, VP41A, VP53A, VP53B, VP51B, VP60A, VP110, VP124, and VP337 [15,28]. Since these envelope proteins form a large infectome complex, the use of a single recombinant protein alone does not completely inhibit viral infection in immune-neutralisation experiments. It is speculated that recombinant proteins of the infectome complex could be used to achieve better protection. However, additional studies are required to explore the mechanisms through which recombinant proteins of the infectome complex are constructed, and how they are combined and administered. This study confirmed that Inno A1 derived from the WSSV infectome complex (particularly the VP53A protein) can improve the immunity of shrimp, reduce disease incidence, and does not affect the diversity of the gut microbiota, thus demonstrating that Inno A1 can be applied in aquaculture. Therefore, this study provides a basis for the better application of feed additives to combat pathogens in shrimp aquaculture.

## Figures and Tables

**Table 1:** List of primers used in this study.

Target Gene	Primer	Sequence (5′-3′)	Usage
Prophenoloxidase (*proPO*)	proPO-F	GAGATCGCAAGGGAGAACTG	qPCR
	proPO-R	CGTCAGTGAAGTCGAGACCA	
Transglutaminase (*TGase*)	TGase-F	CCTCAGGATCTCCTTCACCA	qPCR
	TGase-R	TTGGGAAAACCTTCATTTCG	
Clotting protein (*CP*)	CP-F	TCTTTGCGCAGTTGGTGATC	qPCR
	CP-R	TGAGGTGACCGAGTGCAAAA	
Anti-LPS factor (*ALF*)	ALF-F	CTGTGGAGGAACGAGGAGAC	qPCR
	ALF-R	CCACCGCTTAGCATCTTGTT	
Crustin	Crustin-F	GAGGGTCAAGCCTACTGCTG	qPCR
	Crustin-R	ACTTATCGAGGCCAGCACAC	
Lysozyme	Lyz-F1	GTGGCTTACAACAGCAAGTG	qPCR
	Lyz-R1	CTAGAACGGGAAGACAGAGTTG	
Penaiedin2	PEN2-F	TCGTGGTCTGCCTGGTCTT	qPCR
	PEN2-R	CAGGTCTGAACGGTGGTCTTC	
Penaiedin3	PEN3-F	CACCCTTCGTGAGACCTTTG	qPCR
	PEN3-R	AATATCCCTTTCCCACGTGAC	
Penaiedin4	PEN4-F	GCCCGTTACCCAAACCATC	qPCR
	PEN4-R	CCGTATCTGAAGCAGCAAAGTC	
Superoxidase dismutase (*SOD*)	SOD-F	ATCCACCACACAAAGCATCA	qPCR
	SOD-R	AGCTCTCGTCAATGGCTTGT	
Glutathione peroxidase (*GPx*)	GPx-F	TTTTTCCGTGCAAAAAGGAC	qPCR
	GPx-R	TAATACGCGATGCCCCTAAC	
Toll receptor (*Toll*)	Toll-F1	TGCTGTTGAGCATCAGTGAATA	qPCR
	Toll-R1	AGAACCGCAAACAGGAGAAG	
Elongation factor 1-α (*EF1-α*)	EF1α-F	GGAGATGCACCACGAAGCTC	qPCR
	EF1α-R	TTGGGTCCGGCTTCCAGTTC	
Dscam	Ds-Real-4573F	ACAAGCCAAGGCACCAGACT	qPCR
	Ds-Real-4635R	GTTGCCTGTTGGGCTCACTT	
16S V3-V4	16s-F	TCGTCGGCAGCGTCAGATGTGTATAAGAGSCAG	high-throughput amplicon sequencing
	16s-R	GTCTCGTGGGCTCGGAGATGTGTATAAGAGACA	

**Table 2:** Pathogen detection in the collected samples from the B, C, and P groups. The ratios represent infected specimens/collected specimens.

Group		Specimen	WSSV	*V. parahaemolyticus*		EHP
				AHPND	Non-AHPND	
Before	B group	shrimp	2/14	4/14	2/14	13/14
After		shrimp	0/10	0/10	1/10	9/10
	C group	pool water *	0/1	0/1	0/1	0/1
		Sediment *	0/1	0/1	0/1	0/1
		shrimp	0/15	0/15	0/15	14/15
	P group	pool water *	0/1	0/1	0/1	0/1
		Sediment *	0/1	0/1	0/1	0/1

* Pool water and sediment samples were collected at three different locations of the pond, then pooled together for the pathogen detection.

**Table 3:** Shrimp farming production at the end of the feeding trial of the basal feed (C group) or Inno A1-containing feed (P group). The number of shrimp used for average weight and length measures were 43 and 100 for the C and P groups, respectively.

	C Group	P Group
Total weight harvest (g)	275	11,664
Total shrimps harvest (calculated by average weight)	43	1111
Survival rate (%)	2.15%	55.54%
Sample average weight (g)	6.4 ± 3.1	10.5 ± 3.2
Average length (cm)	9.5 ± 1.6	11.8 ± 1.2

**Table 4:** Differential abundance analysis of bacterial profiles.

Phylum	Classification	#ASV	Maximum Fold Changes
	P > C		
Bacteroidetes	Bacteroidia, Flavobacteriaceae	8	1302.7
Proteobacteria	Alphaproteobacteria, Rhodobacteraceae	20	980.9
	Alphaproteobacteria, Rhizobiaceae	2	187.6
	Gammaproteobacteria, Gammaproteobacteria Incertae Sedis	6	867.2
	Gammaproteobacteria, Alteromonadales	2	718.5
	Gammaproteobacteria, Chromatiales	3	359.3
	Gammaproteobacteria, Cellvibrionales	2	123.1
	Gammaproteobacteria, Steroidobacterales	2	116.2
	Gammaproteobacteria, Others	4	96.5
	Deltaproteobacteria, Oligoflexales	2	231.7
Firmicutes	Erysipelotrichia, Erysipelotrichaceae	1	269.5
Actinobacteria	Acidimicrobiia, Ilumatobacteraceae	3	158.0
Patescibacteria		3	118.4
Planctomycetes	Planctomycetacia, Pirellulaceae	1	68.2
	C > P		
Proteobacteria	Gammaproteobacteria, Gammaproteobacteria Incertae Sedis	1	914.7
	Gammaproteobacteria, Alteromonadales	3	217.6
	Gammaproteobacteria, Vibrionales	2	166.5
	Gammaproteobacteria, Chromatiales	2	129.1
	Gammaproteobacteria, Steroidobacterales	2	114.4
	Gammaproteobacteria, Others	3	109.7
	Alphaproteobacteria, Rickettsiales	2	310.3
	Alphaproteobacteria, Others	2	81.5
	Alphaproteobacteria, Rhodobacteraceae	1	79.0
	Deltaproteobacteria, Bdellovibrionales	3	236.2
Patescibacteria		6	647.7
Bacteroidetes	Bacteroidia, Marinilabiliaceae	2	565.7
	Bacteroidia, Cyclobacteriaceae	1	65.7
Dadabacteria		1	310.3
Chloroflexi		1	75.6
Planctomycetes		1	36.2
Firmicutes	Clostridia, Defluviitaleaceae	1	30.3
Chlamydiae		1	28.0

## Data Availability

The authors confirm that the data supporting the findings of this study are available within the article.

## Supplementary Materials

The following supporting information can be downloaded at: https://www.mdpi.com/article/10.3390/v14071353/s1, Figure S1: Non-metric multidimensional scaling plot of samples for ASV dataset. Each point is a sample (B group (purple), C group (green), and P group (red)), and the distance between points is proportional to the multivariate difference between samples.
