# Supplementary material for: Applying Modified VP53A Recombinant Protein as an Anti-White Spot Syndrome Virus Biological Agent in Litopenaeus vannamei Farming
Source: Viruses. 2022 Jun 21;14(7):1353. doi: 10.3390/v14071353 (PMC9324474; doi:10.3390/v14071353)

viruses

Supplementary Figure S1. Non-metric multidimensional scaling plot of samples for ASV dataset. Each point is a sample [B group (purple), C group (green), and P group (red)], and the distance between points is proportional to the multivariate difference between samples.

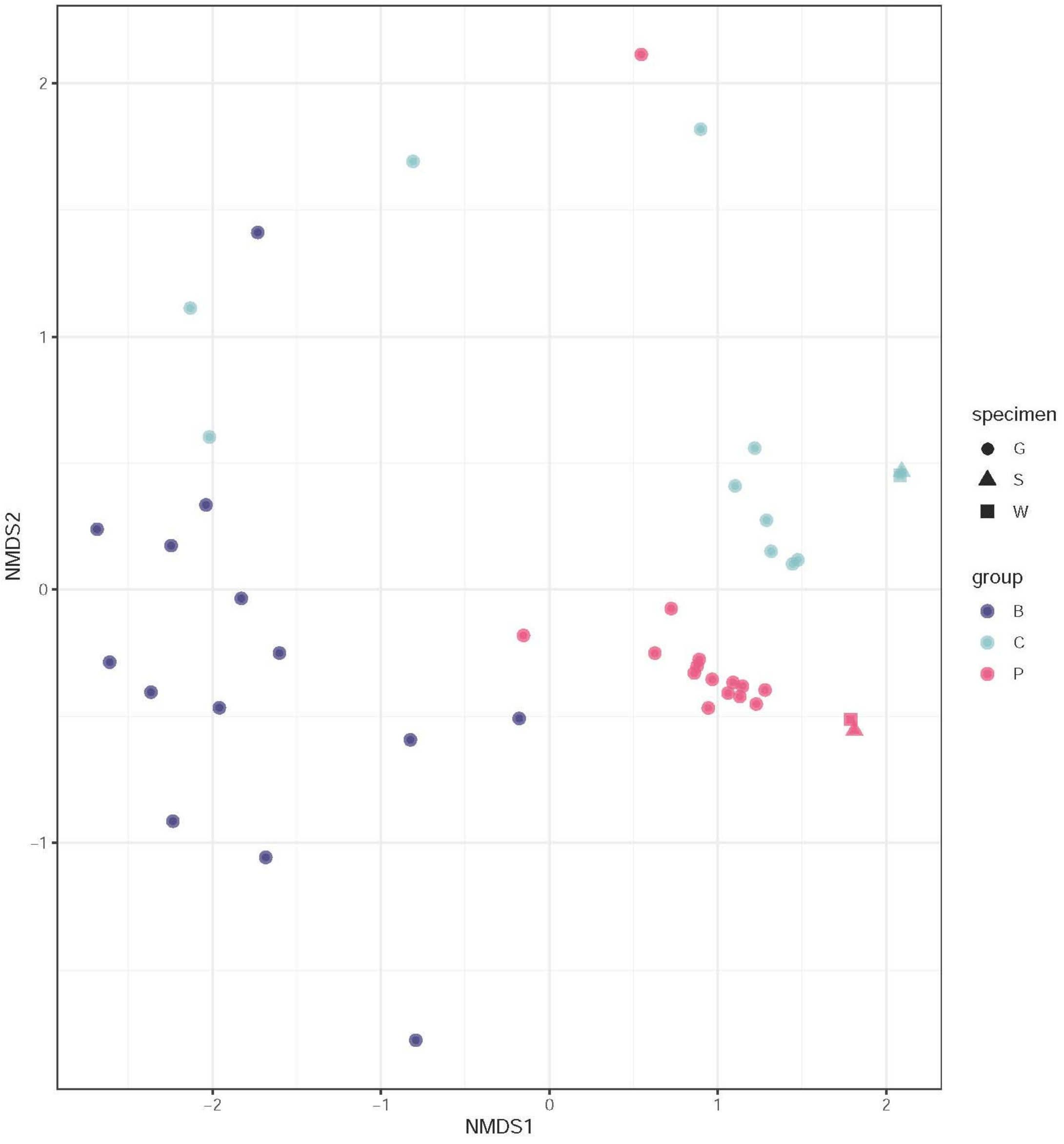

Supplement: Supplementary file 1 [file viruses-14-01353-s001.zip › viruses-1750185-supplementary.pdf]
